# Reactive astrocytes mediate TSPO overexpression in response to sustained CNTF exposure in the rat striatum

**DOI:** 10.1186/s13041-023-01041-x

**Published:** 2023-07-05

**Authors:** Kelly Ceyzériat, Alekos Nicolaides, Quentin Amossé, Christine Fossey, Thomas Cailly, Frédéric Fabis, Valentina Garibotto, Carole Escartin, Benjamin B. Tournier, Philippe Millet

**Affiliations:** 1 Division of Adult Psychiatry, Department of Psychiatry, University Hospitals of Geneva, Geneva, Switzerland; 2 Department of Psychiatry, University of Geneva, Avenue de la Roseraie, 64, Geneva, 1205 Switzerland; 3 CIBM Center for BioMedical Imaging, Faculty of Medicine, University of Geneva, Geneva, Switzerland; 4 Normandie Univ, UNICAEN, Centre d’Études et de Recherche sur le Médicament de Normandie (CERMN), Caen, France; 5 Department of Nuclear Medicine, CHU Cote de Nacre, Caen, France; 6 Normandie Univ, UNICAEN, IMOGERE, Caen, France; 7 Institut Blood and Brain @Caen-Normandie (BB@C) Boulevard Henri Becquerel, Caen, 14074 France; 8 Division of nuclear medicine and molecular imaging, Diagnostic Department, Geneva University Hospitals and University of Geneva, Geneva, Switzerland; 9 Université Paris-Saclay, CEA, CNRS, MIRCen, Laboratoire des Maladies Neurodégénératives, Fontenay-aux-Roses, Gif-sur-Yvette, France

**Keywords:** TSPO, CNTF, SOCS3 - astrocytes, Microglia, Endothelial cells, Lentiviral vectors - SPECT, Fluorescence-activated cell sorting to radioligand-treated tissue

## Abstract

The 18 kDa translocator protein (TSPO) is a classical marker of neuroinflammation targeted for in vivo molecular imaging. Microglial cells were originally thought to be the only source of TSPO overexpression but astrocytes, neurons and endothelial cells can also up-regulate TSPO depending on the pathological context. This study aims to determine the cellular origin of TSPO overexpression in a simplified model of neuroinflammation and to identify the molecular pathways involved. This is essential to better interpret TSPO molecular imaging in preclinical and clinical settings. We used lentiviral vectors (LV) to overexpress the ciliary neurotrophic factor (CNTF) in the right striatum of 2-month-old Sprague Dawley rats. A LV encoding for β-Galactosidase (LV-LacZ) was used as control. One month later, TSPO expression was measured by single-photon emission computed tomography (SPECT) imaging using [^125^I]CLINDE. The fluorescence-activated cell sorting to radioligand-treated tissue (FACS-RTT) method was used to quantify TSPO levels in acutely sorted astrocytes, microglia, neurons and endothelial cells. A second cohort was injected with LV-CNTF and a LV encoding suppressor of cytokine signaling 3 (SOCS3), to inhibit the JAK-STAT3 pathway specifically in astrocytes. GFAP and TSPO expressions were quantified by immunofluorescence. We measured a significant increase in TSPO signal in response to CNTF by SPECT imaging. Using FACS-RTT, we observed TSPO overexpression in reactive astrocytes (+ 153 ± 62%) but also in microglia (+ 2088 ± 500%) and neurons (+ 369 ± 117%), accompanied by an increase in TSPO binding sites per cell in those three cell populations. Endothelial cells did not contribute to TSPO signal increase. Importantly, LV-SOCS3 reduced CNTF-induced astrocyte reactivity and decreased global TSPO immunoreactivity (-71% ± 30%), suggesting that TSPO overexpression is primarily mediated by reactive astrocytes. Overall, this study reveals that CNTF induces TSPO in multiple cell types in the rat striatum, through the JAK2-STAT3 pathway in astrocytes, identifying this cell type as the primary mediator of CNTF effects neuroinflammatory processes. Our results highlight the difficulty to interpret TSPO imaging in term of cellular origin without addition cellular analysis by FACS-RTT or quantitative immunostainings. Consequently, TSPO should only be used as a global marker of neuroinflammation.

## Introduction

Neuroinflammation is a common feature to numerous brain diseases. It involves astrocytes and microglial cells that react to environmental changes translated by transcriptomic, proteomic and functional alterations. Among classical markers of neuroinflammation, increased levels of the 18 kDa translocator protein (TSPO) have been reported consistently [1, 2] and active research is still ongoing to better quantify this phenomenon with in vivo molecular imaging (i.e. positron emission tomography [PET]/single-photon emission computed tomography [SPECT]). Multiple radiotracers have been developed to quantify TSPO overexpression *in vivo.* The 1st generation tracers presented non negligible limitations, including low sensitivity and high level of non-specific binding leading to a poor signal-to-noise ratio [3, 4], a characteristic improved by next generations of radiotracers with higher affinity for TSPO [4–6]. The multicellular origin of TSPO also complicates the interpretation of the signal measured. Indeed, TSPO overexpression has for a long time been considered to occur only in microglial cells but recent studies showed that astrocytes, neurons and endothelial cells could also contribute to increased TSPO PET/SPECT signal, depending on the stimulus [1]. For example, we recently showed that in the frontal cortex of Alzheimer’s disease subjects, the most frequent neurodegenerative disease, both astrocytes and microglia overexpress TSPO [7], and TSPO increase in astrocytes could even appear earlier than in microglia [8]. In brain tissue of epileptic subjects, neurons have been shown to contribute to TSPO overexpression in concert with microglia [9]. Acute inflammation induced either by single injection of ciliary neurotrophic factor (CNTF) or lipopolysaccharide (LPS) did not induce the same changes in TSPO-expression. In the first case, both astrocytes and microglia contributed to TSPO overexpression whereas only microglial cells overexpressed TSPO with LPS [7]. Consequently, caution should be taken when interpreting TSPO imaging in preclinical and clinical settings.

A previous study using lentiviral vectors (LV) to induce sustained CNTF expression in the brain showed that this cytokine leads to TSPO overexpression by reactive astrocytes [10]. Nevertheless, the contribution of other cell types was not quantified in this simplified model of inflammation. Using the fluorescence-activated cell sorting to radioligand-treated tissue (FACS-RTT) method that we recently developed [7, 8, 11, 12], we aimed to better characterize the impact of CNTF on TSPO expression by astrocytes but also by other brain cells. Moreover, CNTF is known to directly activate molecular pathways, such as the mitogen-activated protein kinases [13] or the Janus kinase-signal transducer - activator of transcription 3 (JAK-STAT3) [14]. In astrocytes, JAK-STAT3 pathway activation could directly be linked to TSPO overexpression as the TSPO promoter bears a STAT3 binding site [15]. To validate this hypothesis, we co-injected a LV-CNTF and a LV encoding for SOCS3, an inhibitor of JAK-STAT3 pathway known to inhibit astrocyte reactivity in different pathological conditions [16–18].

## Methods

**Animals and stereotaxic injections.** Male Sprague-Dawley rats were housed with food and water *ad libitum*, in a 12 h light-dark cycle. All experimental procedures were approved by the Ethics Committee for Animal Experimentation of the Canton of Geneva, Switzerland or the local ethics committee (CETEA N°44) and submitted to the French Ministry of Education and Research (Approval APAFIS#4554-2016031709173407). Data are reported in accordance with Animal Research: Reporting In vivo Experiments (ARRIVE) guidelines.

**Lentiviral vector production.** Self-inactivated lentiviral vectors containing the mouse phosphoglycerate kinase (PGK) I promoter were produced and pseudotyped with the G-protein of the vesicular stomatitis virus to target neurons (LVn). LVn encoding the ciliary neurotrophic factor (LVn-CNTF) with the export sequence of Ig was used to mainly induce astrocyte reactivity [10, 19]. LVn encoding the beta-galactosidase gene (LVn-LacZ) was used as control. Other lentiviral vectors were pseudotyped with the G-protein of the Mokola lyssavirus to infect astrocytes (LVa) and contained four copies of the target sequence of the neuronal miRNA124 to prevent transgene expression in neurons. LVa encoding the suppressor of cytokine signaling 3 (LVa-SOCS3) under the PGK promoter was used to specifically inhibit astrocyte reactivity [14, 17]. LVa encoding for the blue fluorescent protein (LVa-BFP) in the same backbone was used as control. LVa encoding for the green fluorescent protein (LVa-GFP) was used to visualize infected astrocytes. Lentiviral vectors were diluted in 1% BSA/PBS 0.1 M at a final concentration of 100 ng p24/ml. The production and titration of these lentiviruses were described previously [20].

**Stereotaxic surgery.** Two-month-old Sprague-Dawley rats were simultaneously injected in the left striatum with LVn-LacZ (100 ng of p24) and in the right striatum with LVn-CNTF (100 ng of p24) (Cohort 1; n = 11). Rats were anesthetized with 2% isoflurane under local anesthesia (Lidocaine 7 mg/kg) at the injection site and received analgesic before and after injection (Buprenorphine 0.05 mg/kg). The stereotaxic coordinates used were the following: anteroposterior (AP): +0.5 mm and lateral (L): ±3 mm from bregma; ventral (V): -5 mm from the dura. After the injection of 2 µl of LVn at a rate of 0.2 µl/min, the needle was left in place for 2 min before withdrawal. Imaging experiments were then performed 1 month post injection. In a second cohort, 2-month-old Sprague Dawley rats were anesthetized with a mixture of ketamine (75 mg/kg) and xylazine (10 mg/kg) and injected with LVn-CNTF (41 ng of p24) + LVa-BFP (204 ng of p24) + LVa-GFP (55 ng of p24) in the left striatum and LVn-CNTF (41 ng of p24) + LVa-SOCS3 (204 ng of p24) + LVa-GFP (55 ng of p24) in the right striatum (Cohort 2; n = 3). The stereotaxic coordinates used were the following: anteroposterior (AP): +0.5 mm and lateral (L): ±3 mm from bregma; ventral (V): -4 mm from the dura. Three microliters of LV mix were injected at a rate of 0.25 µl/min in each striatum. Animals were euthanized after 2 months.

**[**^**125**^**I]CLINDE synthesis.** The CLINDE tributyltin precursor (100 µg) in acid acetic (100 µl) was incubated at 70 °C for 20 min with 5–10 mCi Na^125^I (PerkinElmer) and 37% peracetic acid (5 µl). After purification using a reversed-phase column, [^125^I]CLINDE, a TSPO specific radiotracer, was concentrated using a Sep-Pak C18 cartridge in 95% acetonitrile (ACN). Then, ACN was evaporated and [^125^I]CLINDE was dissolved in saline.

**SPECT imaging and image processing.** One month post injection, rats were anesthetized (2% isoflurane) and received a tail-vein injection of [^125^I]CLINDE (28.30 ± 0.92 MBq). Animals were placed in the U-SPECT-II scan (n = 4; MiLabs). A dynamic SPECT scan (60 × 1-min frames) was acquired starting with radiotracer injection. SPECT images were reconstructed using a P-OSEM (0.4 mm voxels, 4 iterations, 6 subsets) method with a radioactive decay correction. Dynamic images were manually co-registered to the T2 Schwartz template of the small animal molecular imaging toolbox (SAMIT [21]) using PMOD software (version 4.2, PMOD Technologies Ltd). A factorial analysis correction was applied to reduce noise in SPECT data using Pixies (Apteryx) as previously described [22, 23]. Then, time activity curves were generated from the Px Rat (Schwartz) template using PMOD. Non-displaceable Binding potentials (BP_ND_) values were estimated using the Logan approach [24], with an explicit mask on the signal coming from outside of the brain and whole cerebellum as reference region. BP_ND_ images were flipped to allow statistical parametric mapping using SAMIT and SPM12 software of both hemispheres of the same animal. A paired t-test was realized on smoothed images with an implicit mask (eliminating signal from outside of the brain) and an explicit mask for the left hemisphere. A *p* value threshold of 0.01 was used. A volume-of-interest (VOI)-based analysis on BP_ND_ images was then performed to compare TSPO binding in both striata using the Px Rat (Schwartz) SAMIT template using PMOD.

**Cell dissociation.** After SPECT acquisition, or 1 h post [^125^I]CLINDE injection, animals were euthanized by decapitation while still under isoflurane anesthesia. The brain was removed and dissected on ice to isolate the left and right striata. Samples were then prepared for FACS using the previously described protocol [25]. Briefly, manual mechanic and enzymatic dissociation were performed on tissues followed by a myelin depletion step (Miltenyi biotec). Cells were then suspended in 0.1 M PBS/ 1 mM EDTA/ 1% of BSA buffer and treated with an anti-rat CD32 (1/100; BD Bioscience) for 5 min on ice to block Fc-mediated adherence of antibodies. After a centrifugation at 350 g, 5 min at 4 °C, cells were resuspended with a solution of primary antibodies diluted in 0.1 M PBS/ 1 mM EDTA/ 1% of BSA buffer: APC anti-rat CD11b (1/800; Biolegend), FITC anti-rat CD90 (1/250; Biolegend), PE-Cy7 anti-rat PECAM/CD31 (1/100; Invitrogen) and anti-GFAP-Cy3 (1/15; Sigma) antibodies for 20 min on ice. After a centrifugation at 350 g, 5 min at 4 °C, cells were suspended in 250 µl of buffer for cell sorting.

**Fluorescence activated cell sorting.** Unstained and single stain cells were used to identify positive and negative cells for each antibody, to identify auto-fluorescent cells, to determine the values of compensation (to correct for interference between fluorochromes) and to draw sorting gates for each antibody. Cells were first sorted based on their forward and side scatter from all detected events. Dead cells were excluded using Hoechst (incubated for 10 min on ice at 5 µg/ml; Abcam) staining before cell sorting. First, astrocytes and endothelial cells were sorted based on their fluorescence for GFAP-Cy3 and PE-Cy7-PECAM/CD31 respectively. GFAP^−^/CD31^−^ cells were then sorted based on their fluorescence for FITC-CD90 positive cells (neurons) and APC-CD11b (microglia cells). Cells that were neither auto-fluorescent nor stained with an antibody were not sorted. Cell numbers were determined for each cell population during cell sorting. Samples were sorted on a MoFlo Astrios (Beckman Coulter), and data were analyzed with FlowJo 10.4 (FlowJo LLC).

***Ex-vivo*radioactivity counting**. Radioactive concentrations in rat striatum (before cell dissociation) and in isolated cell types were measured on an automatic γ counting system (Wizard 3”, PerkinElmer). Data are expressed in percentage of the injected dose (% ID)/g of tissue for each animal.

**Immunohistology.** Animals of the second cohort were euthanized with an overdose of sodium pentobarbital and perfused intracardially with 4% paraformaldehyde. Then, brains were extracted and cryoprotected with a 30% sucrose solution. Thirty microns coronal slices were prepared on a freezing microtome and stored at − 20 °C until use for histology. Brain slices were rinsed in PBS 0.1 M, mounted on gelatin slides, and let dry overnight (O/N). After immersing slides into PBS 0.1 M, antigen retrieval was achieved using 0.01 M sodium citrate buffer for 5 min at 100 °C. Slides were cooled for 15 min at room temperature (RT). After a 5 min wash in dH_2_O, slides were immersed into PBS0.1 M and incubated with TSA blocking buffer (Akoya Biosciences) for 1 h at RT. Slides were then incubated O/N at 4 °C with the primary antibodies: anti-GFAP (1/500; Invitrogen), anti-TSPO (1/2000, NP155; kindly provided by Dr Higuchi, National Institutes for Quantum Science and Technology, Chiba, Japan), diluted in the TSA blocking buffer. After washes, slides were incubated with secondary antibodies: anti-mouse Alexa647nm (1/500; Alexa Fluor), anti-rabbit-biotinylated (1/1000; Merck) for 1 h at RT in TSA blocking buffer. Slides were washed and incubated with the SA-HRP antibody (1/100; Akoya Biosciences) for 30 min at RT in TSA blocking buffer. After washes, slides were incubated with the diluted Tyramide reagent Cy3 (1/50; Akoya Biosciences) in 1x Amplification diluent (Akoya Biosciences) for 10 min at RT and washed again. Finally, slides were coverslipped with FluorSave mounting medium.

**Quantification of immunostaining.** Images were acquired using an Axioscan.Z1® (Zeiss) at 10x magnification. The area where SOCS3 decreased GFAP signal was manually delimited and transposed to the contralateral side to quantify the mean grey value of GFAP staining (representing GFAP levels) in both striata using ImageJ. In the same region of interest (ROI), an intensity threshold was applied to quantify the percentage of the ROI positively stained for TSPO (% TSPO^+^ area). Then, a mask was defined on the GFAP^+^ area in the ROI and applied on the TSPO image with the intensity threshold to quantify the TSPO^+^/GFAP^+^ area. Two or three slices were quantified per animal. Data are expressed as a mean of values obtained per animal.

**Statistical analyses.** Radioactivity was expressed as BP_ND_, as a percentage of the injected dose/g tissue weight (%ID/g) or as a percentage of the injected dose/cell (%ID/cell). Normality of residues was validated with the Shapiro-Wilks test. Data were analyzed using a two-tailed paired t-test when comparing left and right striata. Two-way ANOVA (Viral vector and cell type as between factors) and Bonferroni’s post hoc test were used to compare median fluorescence intensity (MFI) values and the number of cells sorted/g of tissue. All analyses were performed using Prism software (Prism 9.4.0, GraphPad). Data are presented as mean ± SEM. For all figures, each point represents individual value per animal.

## Results

### Sustained CNTF exposure leads to an increased TSPO binding in the rat striatum

**Fig. 1:**
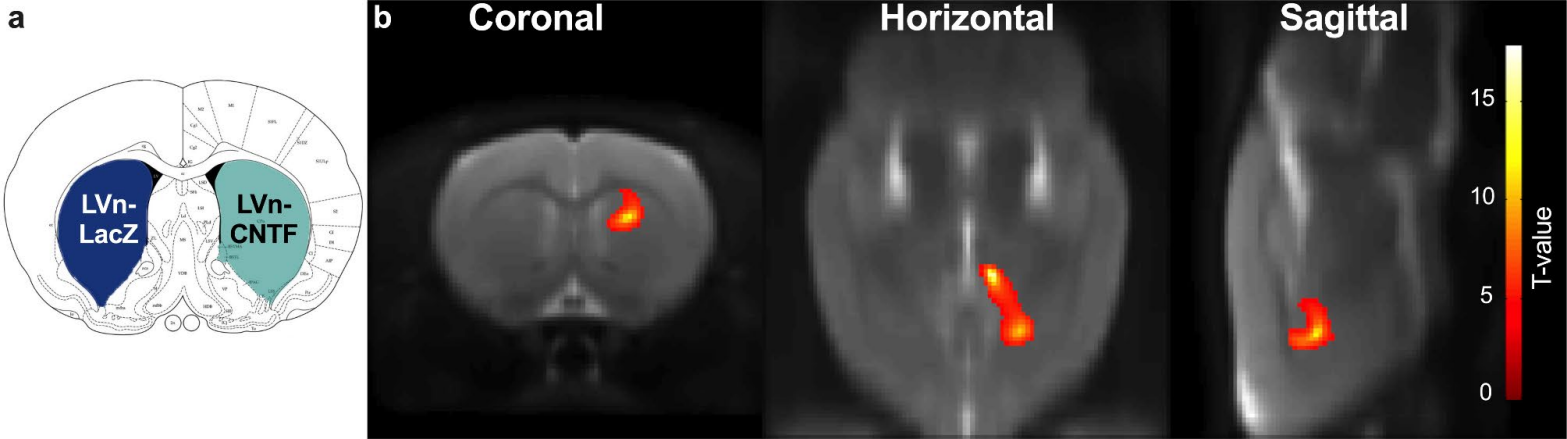
**Voxel-based analysis of TSPO overexpression in the rat striatum after LVn-CNTF injection**. (**a**) Two-month-old rats were injected with LVn-CNTF (inflammation induction) and LVn-LacZ (control) in right and left striatum, respectively. (**b**) Hemisphere comparison at the voxel level using statistical parametric mapping (SPM) on [^125^I]CLINDE SPECT BP_ND_ images was performed using the small animal molecular imaging toolbox (SAMIT). Results are plotted in the Schwarz MRI from SAMIT and presented in each plan (coronal, horizontal and sagittal of the right hemisphere). A significant increase of TSPO binding was observed in the right (LVn-CNTF) compared to the left (LVn-LacZ, Control) striatum (n = 4 rats). Colored voxels indicated a difference with a *p* value *< 0.01*

**Fig. 2:**
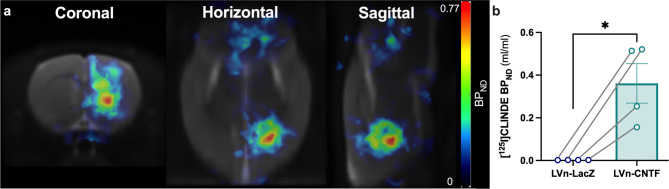
**Volume-based analysis of TSPO overexpression in the rat striatum after LVn-CNTF injection**. (**a**) Average images of [^125^I]CLINDE non-displaceable binding potential (BP_ND_) acquired by single-photon emission computed tomography (SPECT) imaging in 4 rats and co-registered to the MRI atlas in the coronal (left), horizontal (center) and sagittal (right) planes. TSPO binding is increased in the right striatum compared to the left striatum. Scale bar = 0-0.77 BP_ND_. (**b**) Quantification of [^125^I]CLINDE BPnd in the left (LVn-LacZ) and the right (LVn-CNTF) striatum demonstrating a clear overexpression of TSPO after LVn-CNTF injection (n = 4 rats). Paired t-test, * *p < 0.05*

**Fig. 3:**
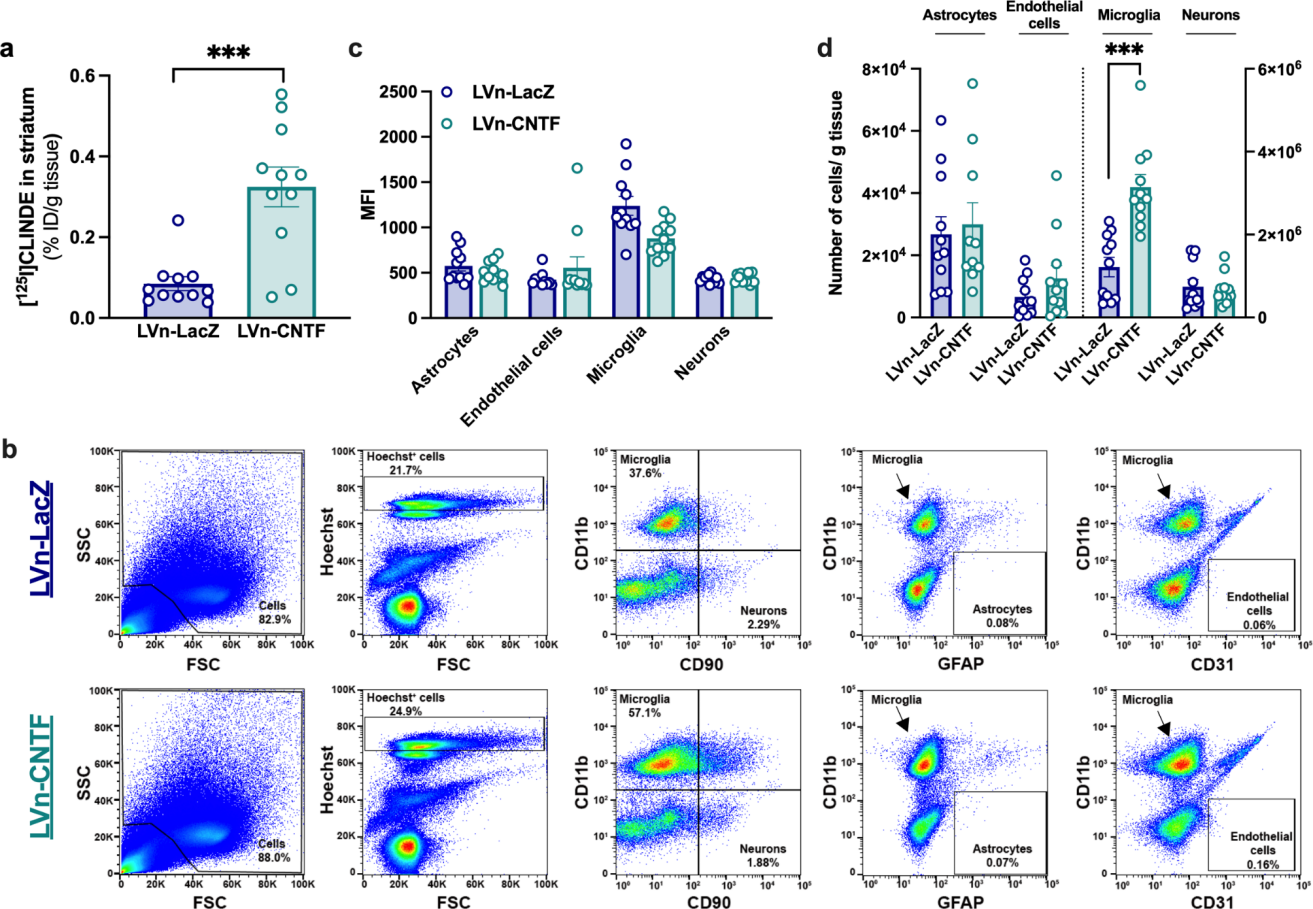
**LVn-CNTF induces TSPO overexpression and microglial cell proliferation in the rat striatum**. (**a**) Quantification of radioactivity (% injected dose (%ID)/g of tissue) in each dissected striatum by gamma-counting (n = 11 rats). A significant increase in TSPO binding is observed in the right striatum injected with LVn-CNTF. Paired t-test, *** *p < 0.001.* (**b**) Representative gates for cell sorting by flow cytometry in the LVn-LacZ hemisphere (upper panel) and the LVn-CNTF hemisphere (lower panel). From left to right, cells were selected based on their granularity and size, following by the selection of alive cells (Hoechst^+^) and singulets (not represented). Microglial cells (CD11b^+^) and neurons (CD90^+^) were selected in GFAP^−^/CD31^−^ double negative cells. The two last plots showed that astrocytes (GFAP^+^) and endothelial cells (CD31^+^) did not contaminate the microglial population. The percentage of alive cells (Hoechst^+^) is expressed in relation to cells. The percentage of each cell population is represented in relation to singlets. (**c**) Median fluorescence intensity (MFI) for each cell type in the right and left striatum measured during cell sorting (n = 11 rats). No difference was observed between hemispheres. Two-way ANOVA (viral vector and cell type as between factors). (**d**) Number of isolated cells normalized by the total number of cells. Numbers of astrocytes and endothelial cells are scaled on the left Y axis, whereas numbers of microglia and neurons are scaled on the right Y axis. An increased number of CD11b^+^ cells is observed in the right striatum compared to the contralateral side, suggesting a proliferation of microglia after LVn-CNTF injection (n = 11 rats). Two-way ANOVA (Viral vector and cell type as between factors) and Bonferroni’s post hoc test, *** *p < 0.001*

Two-month-old rats were injected with LVn-LacZ (control virus) in the left striatum or with LVn-CNTF in the right striatum (Fig. 1a), to induce a TSPO overexpression and mainly an astrocyte reactivity [10, 19]. Voxel-based analysis of [^125^I]CLINDE SPECT BP_ND_ images showed a spot with intense signal in the right striatum injected 1 month before with LVn-CNTF (Fig. 1b; paired t-test, *p < 0.01*). This clear overexpression of TSPO after LVn-CNTF injection was confirmed using a volume-based analysis on BP_ND_ images (t [3] = 3.89, *p = 0.0302*; Fig. 2a, b). The specific overexpression of TSPO binding after LVn-CNTF injection was also confirmed using γ counting in dissected striatum (+ 99.4 ± 0.2%; Fig. 3a; t [10] = 4.975, *p = 0.0006*).

### Astrocytes, microglia and neurons but not endothelial cells participate to the TSPO binding increase in the rat striatum

To determine the cellular origin of TSPO signal measured by in vivo SPECT imaging and ex vivo counting, striata were dissociated and astrocytes, microglia, neurons, and endothelial cells were sorted (Fig. 3b). During sorting, we did not observe a modulation of MFI in the right hemisphere injected with LVn-CNTF compared to the contralateral side, ruling out possible bias in cell sorting efficiency between hemispheres (Fig. 3c; Two-way ANOVA, F_(1,20)_ = 2.317, *p = 0.14* for viral vector main effect, F_(3,60)_ = 40.15, *p = 0 < 0.001* for cell type main effect; F_(3,60)_ = 5.233, *p = 0.003* for viral vector x cell type interaction). However, we observed an increased number of CD11b^+^ cells in the LVn-CNTF-injected striatum compared to the control side (Fig. 3b, d; Two-way ANOVA, F_(1,20)_ = 13.67, *p = 0.001* for the main effect of viral vector, F_(3,59)_ = 96.00, *p < 0.001* for the main effect of cell type, F_(3,59)_ = 22.06, *p < 0.001* for viral vector x cell type interaction. Bonferroni’s post hoc test, *p < 0.001*), suggesting a proliferation of microglial cells after LVn-CNTF injection. No difference was observed for any other cell type marker.

**Fig. 4:**
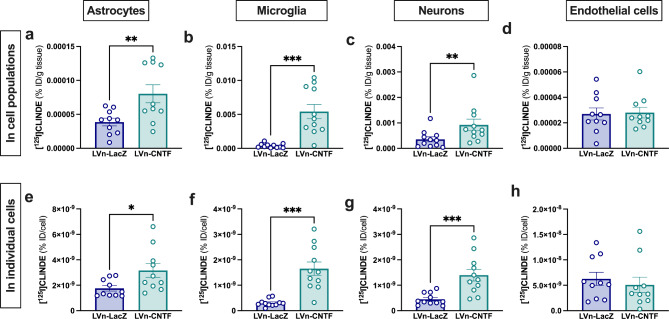
**Cellular origin of TSPO overexpression after LVn-CNTF injection in the striatum of 2-month-old rats**. Radioactivity measurement (% injected dose (% ID)/g of tissue) of [^125^I]CLINDE in astrocytes (**a**), endothelial cells (**b**), microglia (**c**) and neurons (**d**) of the left (LVn-LacZ, Control) and right (LVn-CNTF) striatum, showing an overexpression of TSPO in the right striatum compared to the left striatum in all cell types, except endothelial cells (n = 11 rats). Paired t-test, ** *p < 0.01*, *** *p < 0.001*. Radioactivity per cell (% ID/cell) in the different cell populations: astrocytes (**e**), endothelial cells (**f**), microglia (**g**) and neurons (**h**) of the left (LVn-LacZ, Control) and right (LVn-CNTF) striatum, showing an increase of TSPO binding sites in the LVn-CNTF compared to the LVn-LacZ-injected striatum in all cell populations, except endothelial cells (n = 11 rats). Paired t-test, * *p < 0.05*, *** *p < 0.001*

After sorting, we measured the radioactivity accumulated in the different cell types. Interestingly, we observed an increase of TSPO binding (% ID/g of tissue) in astrocytes (+ 153 ± 62%, Fig. 4a; t [9] = 3.749, *p = 0.0046*), microglia (+ 2088 ± 500%, Fig. 4b; t [10] = 4.900, *p = 0.0006*), neurons (+ 369 ± 117% Fig. 4c; t [10] = 3.289, *p = 0.0082*) in the LVn-CNTF as compared to the LVn-LacZ injected striatum, but not in endothelial cells (Fig. 4d; t [9] = 0.2199, *p = 0.8309*). This result showed that astrocytes, microglia and neurons participated in TSPO increase observed by SPECT imaging. An increase in radioactivity per cell (% ID/cell) was also measured in astrocytes (+ 84 ± 23%, Fig. 4e; t [9] = 3.209, *p = 0.0107*), microglia (+ 448 ± 62%, Fig. 4f; t [10] = 5.585, *p = 0.0002*), neurons (+ 251 ± 58%, Fig. 4g; t [10] = 4.934, *p = 0.0006*), but not in endothelial cells (-19 ± 16%, Fig. 4h; t [9] = 1.423, *p = 0.1885*), showing an increase of the number of binding sites per cell in the three populations, in response to LVn-CNTF injection.

### Reactive astrocytes mediate the TSPO upregulation in response to neuronal CNTF

**Fig. 5:**
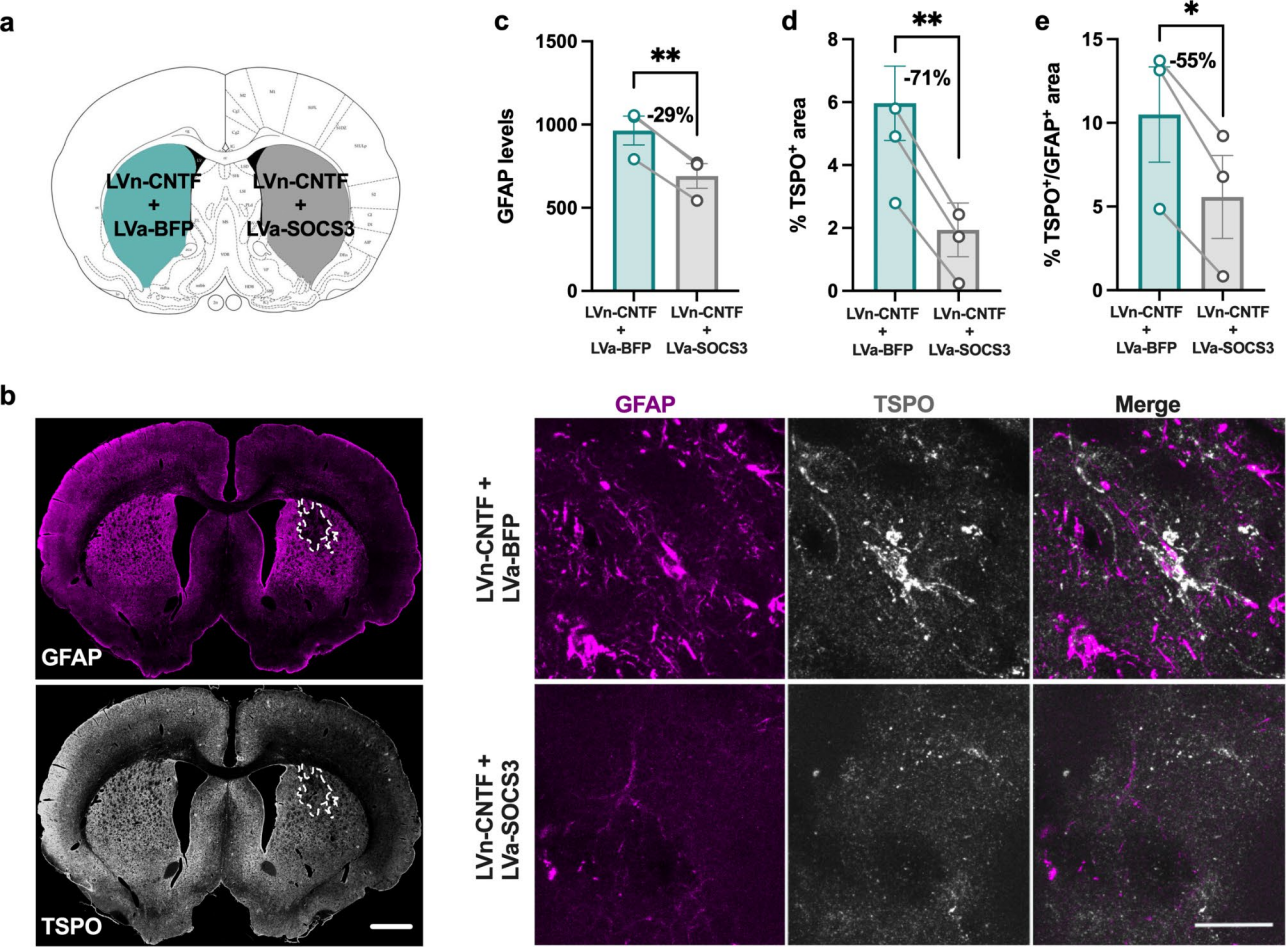
**Inhibition of astrocyte reactivity drastically reduces TSPO signal**. (**a**) A second cohort of rats was injected with LVn-CNTF + LVa-BFP (inflammation induced) and LVn-CNTF + LVa-SOCS3 (astrocyte reactivity inhibited) in the left and right striatum, respectively. (**b**) Representative images of the area devoid of GFAP + astrocytes in the right striatum (left panel). Scale bar = 1 mm. Representative confocal images showing lower GFAP (magenta) and TSPO (grey) expression in presence of SOCS3 (right panel). Scale bar = 25 μm. Quantification of GFAP levels (**c**), TSPO density (**d**, %TSPO^+^ area), and TSPO expression in GFAP^+^ astrocytes (**e**, %TSPO^+^/GFAP^+^ area) in the injected area of both striata (n = 3 rats). The inhibition of the JAK2-STAT3 pathway in astrocytes drastically reduced TSPO expression. Paired t-test, * *p < 0.05*, ** *p < 0.01*

To go further, we investigated molecular pathways related to TSPO overexpression after LVn-CNTF injection. For that, rats were co-injected with LVn-CNTF and LVa-SOCS3 in one striatum and the other with LVn-CNTF and LVa-BFP as control. SOCS3 is the endogenous inhibitor of the JAK-STAT3 pathway and it prevents STAT3-mediated astrocyte reactivity [14, 17] (Fig. 5a). The co-injection of both viruses reduced GFAP expression in the right striatum compared to the contralateral side, showing reduced astrocyte reactivity as reported previously in different animal models [16, 18, 26] (Fig. 5b, c,-29 ± 1.5%,; t [2] = 19.85, *p = 0.025*). A clear reduction of the global TSPO density (% TSPO^+^ area) was also measured in the area with a reduced astrocyte reactivity (-71 ± 30%, Fig. 5b, d; t [2] = 12.20, *p = 0.0066*). The TSPO staining in GFAP^+^ cells was reduced in the striatum injected with LVa-SOCS3 compared to the contralateral side (-55 ± 15%, Fig. 5e; t [2] = 6.933, *p = 0.0202).*

## Discussion

The cellular origin of increased TSPO signal measured by PET/SPECT molecular imaging in conditions of neuroinflammation is still an open question. For many years, TSPO was thought to be a specific marker of reactive microglia. However, recent studies demonstrated that TSPO is not only overexpressed by microglial cells but also by astrocytes, neurons and/or endothelial cells, depending of the pathological context [1, 7, 27, 28]. Indeed, using FACS-RTT, we recently showed that acute inflammation induced either by LPS or CNTF acute brain injection, triggered different cellular patterns of TSPO overexpression [7]. LPS injection in the rat hippocampus caused microglial TSPO overexpression only, without modulation of the number of binding sites per cell, whereas CNTF induced an astrocytic and microglial TSPO upregulation, with increased binding sites per microglia.

Here, we used lentiviral vectors to promote a sustained CNTF overproduction by striatal neurons, inducing multiple molecular and functional reactive changes in astrocytes [10, 19, 29]. Minimal induction of classical markers of reactive microglial cells (IBA1, CD11b, CD68) was observed, suggesting that LVn-CNTF preferentially activates astrocytes. TSPO induction was observed mostly in GFAP^+^ and Vimentin^+^ reactive astrocytes, but some CD11b^+^ microglia were also found to express TSPO. However, the relative TSPO expression between astrocytes and microglia was not quantified [10]. Therefore, in this study, we aimed to provide a quantitative assessment of TSPO relative expression among the different brain cell types. By in vivo SPECT imaging, we observed a higher TSPO binding after LVn-CNTF injection in the striatum than in the contralateral side injected with the control LVn-LacZ. This TSPO increase was confirmed by the more sensitive ex vivo γ counting. Interestingly, using FACS-RTT, we showed that in addition to astrocytes both microglia and neurons were involved in this TSPO upregulation. We found that TSPO overexpression was higher in CD11b^+^ microglia (+ 2088%) or in CD90^+^ neurons (+ 369%) than in GFAP^+^ astrocytes (+ 153%). An increased TSPO binding sites per cell, representing a higher TSPO expression per mitochondria or an increased number of mitochondria per cell, was also measured in astrocytes, microglia and neurons. However, these results do not allow concluding on the respective contribution of the different cell types to the global TSPO signal. Indeed, it will depend on the proportion of each cell type in the brain but also on the cell marker used to identify cell populations (i.e. GFAP^+^ astrocytes only represent a fraction of astrocytes in the striatum). The choice of an anti-GFAP antibody is therefore one limitation of this study, as it reduces the quantity of astrocytes sorted. A second limitation of this study is the impossibility to directly compare the absolute value of [^125^I]CLINDE levels between cell populations because it depends on cell sorting efficacy, which is influenced by the affinity of each antibody to the targeted cells and by the intensity of the fluorochrome used.

**Fig. 6:**
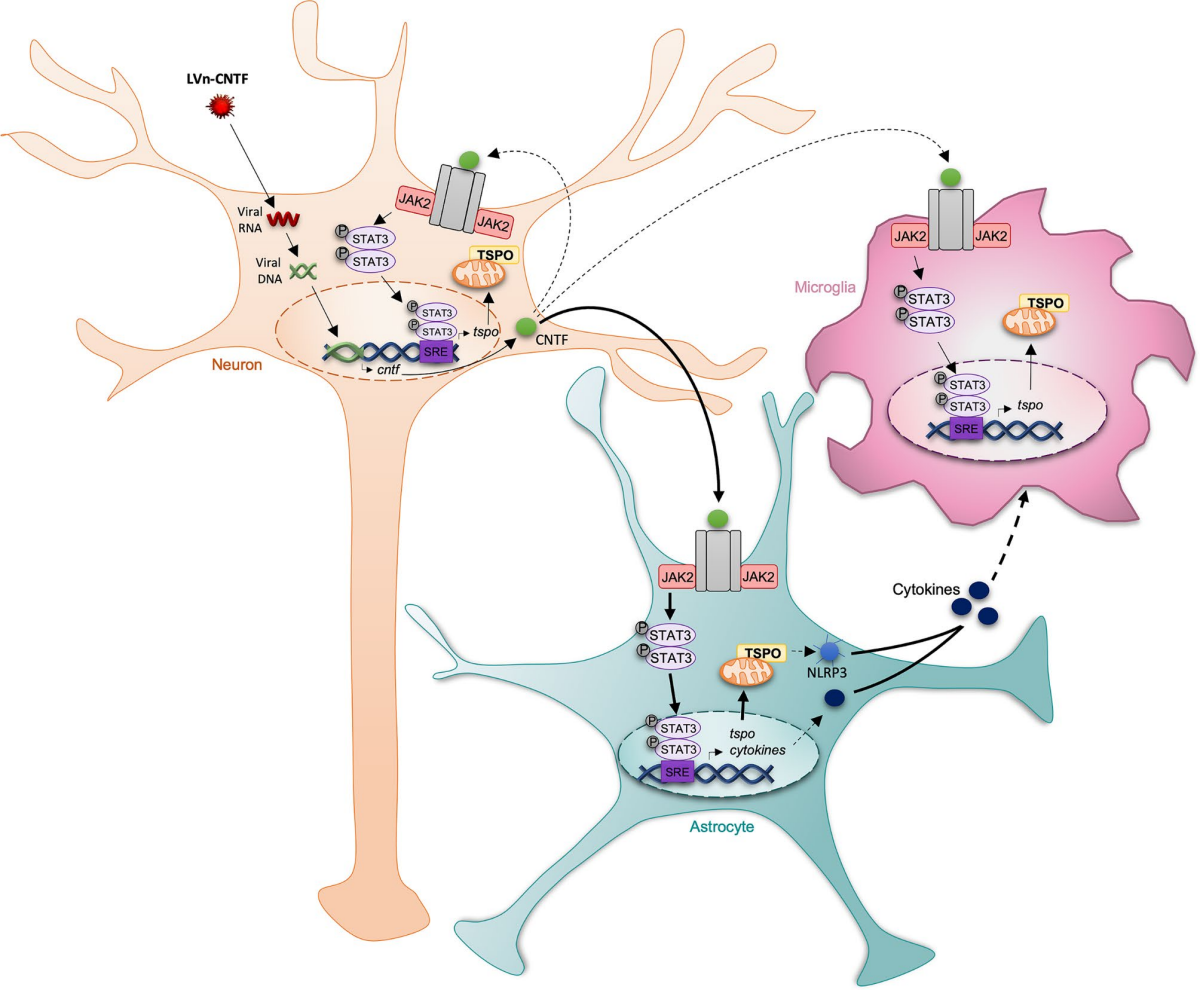
**Potential mechanisms involved in TSPO overexpression in response to sustained CNTF exposure**. Neurons were infected by a lentiviral vector (LVn) encoding for CNTF. CNTF was then released and induced a TSPO overexpression in astrocytes, microglia and neurons. Astrocytes are the first mediators of this response, through the JAK2-STAT3 pathway. The TSPO overexpression in microglia may be due to the release of cytokines by astrocytes in the extracellular space. Nevertheless, CNTF could also be directly responsible of a part of TSPO overexpression in microglia and neurons as both cell type present CNTF receptors. The dotted arrows represent hypothetical mechanisms not demonstrated in this study. The thick arrows highlight the major mechanisms involved in TSPO overexpression

To go further, we aimed to identify the molecular pathways involved in the TSPO up-regulation in response to LVn-CNTF. The TSPO promoter presenting a STAT3 binding site [15], LVn-CNTF was co-injected with an LVa-SOCS3 to inhibit the JAK-STAT3 pathway specifically in astrocytes as demonstrated in other pathological models [16–18]. Reduced astrocyte reactivity was confirmed in our experimental condition by decreased GFAP levels in the striatum and was accompanied by a drastic decrease of TSPO levels. The significant SOCS3-mediated reduction in TSPO expression demonstrates the involvement of the JAK-STAT3 pathway in CNTF-mediated TSPO overexpression (Fig. 6). It also shows that reactive astrocytes are the first mediators of TSPO signal increase.

Consequently, TSPO overexpression in microglia suggests a fine communication between astrocytes and microglial cells that could induce TSPO overexpression in both cell types, with an induction of microglia proliferation as already published in response to acute CNTF protein injection [30] or a sustained overexpression, as suggested here by the increased number in CD11b^+^ microglial cells after LVn-CNTF injection. This increase of CD11b^+^ cells was not due to a bias in cell sorting as the MFI of CD11b was unchanged in the hemisphere injected with LVn-CNTF. It suggests a reduction of CD11b levels per microglia, but more studies are necessary to better understand this effect. The crosstalk between astrocytes and microglia is well described, particularly through the liberation of specific cytokines [31]. The activation of the JAK-STAT3 pathway by CNTF [14] could induce cytokine production by astrocytes, that could then be released and activate microglial TSPO overexpression (Fig. 6). A recent in vitro study also suggested a role of TSPO in the activation of the nucleotide-binding domain-like receptor protein 3 (NLRP3) inflammasome in microglia and consequently the induction of pro-inflammatory cytokine release [32], such as interleukin (IL)-1β and Il-18 [33]. NLRP3 inflammasome is also observed in reactive astrocytes [34] and thus could correspond to a mechanism linking TSPO overexpression in CNTF-induced reactive astrocytes and neighboring microglial cells. CNTF being produced by neurons and released in the cellular environment in our model, it could also have a direct effect on microglial cells [35], although through the JAK2-STAT3 pathway for example [36, 37] (Fig. 6). However, as previous results failed to detect significant induction of classical markers of reactive microglia following LVn-CNTF injection [10], it suggests that TSPO induction is a sensitive marker of mild microglial response to environmental changes. A recent study also suggests that mechanisms involved in TSPO overexpression are different between animal models and human brains. Indeed, authors suggest that TSPO might serve as a marker of microglial density rather than their reactive state in response to inflammation stimuli in human [38]. The use of FACS-RTT in those cases could allow to assess the amount of TSPO per cells more precisely.

CNTF could also have direct effects within neurons. Indeed, neurons are sensitive to CNTF as they express CNTF receptors [39, 40], activating the expression of downstream targets such as STAT3 [27, 41]. Therefore, the activation of CNTF signaling in neurons could mediate TSPO transcription by those cells (Fig. 6).

Finally, endothelial cells have been shown to be involved in TSPO modulation in a model of acute inflammation induced by an adeno-associated virus encoding the tumor necrosis factor gene [42] or in schizophrenia [43]. In our model, endothelial cells did express TSPO but they were not involved in its signal increase. Indeed, neither the global TSPO binding nor the number of TSPO binding site per cell were changed in endothelial cells. These observations confirmed our previous results with acute CNTF injection [7] or in other pathological contexts [1, 7, 8, 28]. Consequently, this study shows the complex, multi-cellular responses regarding TSPO induction in inflammatory conditions. Molecular imaging with TSPO ligands can only conclude on the cellular mechanisms involved, when coupled to FACS-RTT or quantitative imaging.

Overall, our study extends the understanding of the cellular origin of TSPO signal in a model of chronic inflammation induced by CNTF, with the involvement of astrocytes, microglia and neurons. Moreover, we showed that reactive astrocytes are the major mediators of this TSPO overexpression though the JAK-STAT3 pathway. Overall, this study supports the use of TSPO molecular imaging as a global approach to monitor neuroinflammation non-invasively, but without cellular resolution unless complementary quantitative approaches are used to resolve the specific cellular origin of TSPO increase.

## Data Availability

The datasets used and/or analyzed during the current study are available from the corresponding author on reasonable request.

## List of Abbreviations

ACN: Acetonitrile
BFP: Blue fluorescent protein
BP_ND_: Non-displaceable Binding potential
CNTF: Ciliary neurotrophic factor
FACS: Fluorescence activated cell sorting
FACS-RTT: Fluorescence-activated cell sorting to radioligand-treated tissue
GFP: Green fluorescent protein
ID: Injected dose
IL: Interleukin
JAK-STAT3: Janus kinase-signal transducer and activator of transcription 3
LacZ: β-Galactosidase
LPS: Lipopolysaccharide
LV: Lentiviral vectors
LVn: Lentiviral vectors targeting neurons
LVa: Lentiviral vectors targeting astrocytes
NLRP3: Nucleotide-binding domain-like receptor protein 3
O/N: Overnight
SAMIT: Small animal molecular imaging toolbox
SOCS3: Suppressor of cytokine signaling 3
SPECT: Single-photon emission computerized tomography
TSPO: 18 kDa translocator protein
VOI: Volume-of-interest
